# Machine Learning for Separating Dopamine and Octopamine Electrochemical Signals in Drosophila

**DOI:** 10.1021/acs.analchem.5c04155

**Published:** 2026-01-02

**Authors:** Cheonho Park, B. Jill Venton

**Affiliations:** Department of Chemistry, 2358 University of Virginia, P.O. Box 400319, Charlottesville, Virginia 22904, United States

## Abstract

*Drosophila melanogaster*, the fruit fly, uses the neurotransmitters dopamine and octopamine to mediate learning, enabling adaptive behaviors such as reward seeking and punishment avoidance. Their colocalization in the mushroom bodies makes it challenging to study their individual contributions. Fast-scan cyclic voltammetry allows subsecond monitoring of neurotransmitter dynamics, but simultaneous detection of dopamine and octopamine remains difficult due to overlapping oxidation and reduction peaks in their voltammograms. Traditional signal separation methods, such as principal component regression, assume fixed voltammogram shapes across time. However, this assumption fails for octopamine, which exhibits time-varying voltammograms due to secondary oxidation processes at the same potential as dopamine oxidation. In this study, we use a deep learning-based regression approach that analyzes color plots to separate dopamine and octopamine signals collected in *Drosophila*. Using the distinct primary oxidation peak of octopamine as input, a modified *U*-Net architecture was trained as a regression model to predict the secondary oxidation peak and subtract it from the dopamine-octopamine mixture to isolate dopamine contributions. The method achieved normalized root-mean-square errors of 0.06 for dopamine and 0.08 for octopamine, calculated against ground truth components from computationally generated mixtures. Thus, estimation errors are under 10% and there is reliable signal separation. Applications to experimentally measured mixtures demonstrate accurate signal decomposition, with the predicted dopamine and octopamine concentrations showing strong agreement (*r* = 0.93, CCC = 0.93) in scatter plot analysis. Thus, machine learning provides a robust framework to deconvolute overlapping electrochemical signals from octopamine and dopamine, facilitating simultaneous neurochemical detection.

## Graphical abstract

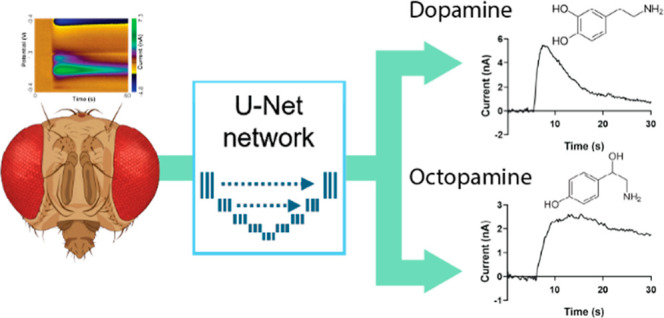

## Introduction

For decades, *Drosophila melanogaster*, the fruit fly, has served as a key model in neuroscience due to its genetic accessibility and well-characterized behaviors.^1–3^ Dopamine and octopamine play central roles in memory and learning in *Drosophila*, with dopamine primarily associated with aversive learning and octopamine with appetitive learning.^4,5^ These neurotransmitters are released quickly in response to environmental cues, highlighting the need for measurement techniques capable of capturing their rapid dynamics.^6,7^ The colocalization of dopaminergic and octopaminergic terminals in the mushroom bodies suggests functional overlap and interaction, making it essential to distinguish their signals when investigating synaptic plasticity and behavior.^8–10^

Fast-scan cyclic voltammetry (FSCV) has been widely used to measure fast neurotransmitter events.^11–13^ FSCV is an electrochemical method that enables subsecond detection of neurotransmitters by applying a rapid scan rate (typically 400 V/s at 10 Hz), allowing real-time monitoring of chemical fluctuations in the brain. Traditionally, FSCV has been used for dopamine detection.^11^ However, with growing interest in octopamine’s role in modulating behavior and reward learning, our group has applied FSCV to study octopamine dynamics in *D. melanogaster*.^14–17^ Simultaneous measurement of dopamine and octopamine using FSCV presents significant challenges because of the overlap of their redox peak potentials, which are commonly used to identify analytes in a voltammogram. Octopamine and dopamine are structurally similar, as octopamine is a phenolamine with a hydroxy group on the side chain (similar to norepinephrine which is its catecholamine equivalent^18^), while dopamine is a catecholamine with no hydroxy group on the side chain.^19^ This challenge is compounded by the redox behavior of octopamine, which includes two oxidation peaks and one reduction peak. The primary oxidation peak, near 1.1 V, corresponds to the initial oxidation of the molecule, but there are further oxidation of the electrochemical byproducts that result in a secondary oxidation peak, near 0.7 V, which is also the primary oxidation peak potential for dopamine. The secondary peak for oxidation changes over time and last longer than the primary peak, so the voltammogram changes over time. Thus, methods are needed to separate the two electrochemical signals in order to monitor them simultaneously.

Several strategies have been developed to address peak overlap in FSCV.^20,21^ One common approach involves modifying the FSCV waveform.^22–27^ In previous studies, our group proposed parameter adjustments to the conventional dopamine waveform (400 V/s, −0.4 to 1.3 V) to better distinguish octopamine.^14,15^ For example, increasing the scan rate to 600 V/s and shifting the holding potential to 0.1 V suppressed octopamine’s secondary oxidation peak. Alternatively, reducing the scan rate to 100 V/s introduced a potential shift between oxidation peaks, which improved their electrochemical resolution. Despite some success, these waveform modifications involve trade-offs of lowering sensitivity.^13,22^ Another approach to separate neurotransmitter signals is the use of machine learning, particularly regression-based methods that estimate voltammogram patterns and their associated concentration profiles. Principal component regression (PCR) is most commonly used to distinguish peaks, which combines dimensionality reduction with linear regression.^20,28–31^ PCR can separate overlapping signals such as dopamine and pH changes and has also been applied to other neurotransmitter combinations.^20^ The method decomposes the three-dimensional FSCV data set, which consists of current, voltage, and time, into two matrices: one capturing the voltammogram shape and the other capturing concentration changes over time.^28^ However, PCR assumes that each analyte has a stable voltammogram over time, which does not hold for octopamine, where the two oxidation peaks vary with time.^21,32^ This limitation highlights the need for alternative strategies to handle time-varying voltammetric features. A previous study from our group addressed the challenge that adenosine, like octopamine, exhibits time-dependent changes in its voltammogram, complicating signal separation.^33^ A pattern-matching method using structural similarity index was employed, in which signals were compared to a set of representative adenosine color plots, each consisting of a time series of voltammograms. By treating these time series as images and leveraging structural similarity across time, this approach successfully captured the dynamic voltammetric features of adenosine.^33^

The goal of this study is to separate octopamine and dopamine signals using a deep learning–based regression approach that treats voltammetric data as two-dimensional images. We selected the *U*-Net architecture because its encoder–decoder design with skip connections allows efficient extraction of both global and localized spatial features, which is ideal for capturing the partially overlapping voltammetric patterns of dopamine and octopamine. Moreover, unlike traditional approaches such as principal component regression that operate on 1D voltammogram vectors, our study adopts a 2D image-based representation of voltammetric data, which consists of time-voltage–current. Therefore, *U*-Net is particularly well-suited for such data, as it was originally developed for biomedical image segmentation and excels in learning structured patterns from spatial matrices. Specifically, the primary oxidation peak of octopamine at approximately 1.1 V is used as input, and the secondary oxidation peak is the output to train a regression network. Since the primary peak is distinct and does not overlap with dopamine, the model can estimate the secondary oxidation component and subtract it from the mixed signal, allowing a prediction of the pure concentration vs trace for dopamine. The machine learning method works well for real mixtures of octopamine and dopamine that are applied to the electrode in a *Drosophila* brain. While we test this machine learning method for dopamine and octopamine, it could be broadly applied in the future to other overlapping neurotransmitter signals.

## Experimental Section

### *D. melanogaster* Brain Tissue Preparation

5- to 10 day-old adult fruit flies, were kept in an ice-filled box for 5 min, then transferred from the vial to a chilled Petri dish and further anesthetized for 1 min.^7^ The brain was isolated in chilled dissecting buffer, and the glial sheath carefully removed using sharp tweezers. The extracted brain was positioned with the anterior side up and affixed to the Petri dish floor with WormGlu (GluStitch Inc., British Columbia, Canada), a biocompatible adhesive designed for aqueous environments. For data acquisition, a CFME (carbon–fiber microelectrode) was positioned at either the heel or medial tip of the MB (mushroom bodies) using a GFP marker to guide target localization.^7^ A pulled glass capillary pipet, with a tip diameter of approximately 10 μm, was filled with the desired neurotransmitter solution and positioned approximately 10–15 μm from the CFME tip. After a 15 min equilibration period, a Picospritzer III (Parker Hannifin, Fairfield, NJ) was used to pressure-inject the neurotransmitter solution into the brain tissue. The injection pressure was fixed at 10 psi, and the injection duration was set to approximately 250 ms, depending on the in situ measured amount of neurotransmitter. The injected volume was approximately 2 nL. Additionally, to vary the concentration, the baseline injection volume was adjusted to obtain data at five different concentration levels. A new capillary was used for each neurotransmitter to avoid cross-contamination. A DS-Qi2 monochrome CMOS camera and NIS-Elements BR imaging software (Nikon Instruments, Melville, NY) were used to capture images and perform distance quantification within the brain.

### Neurotransmitter Injection Experiment

Aqueous solutions of dopamine and octopamine were injected into extracted fruit fly brains and measured using FSCV. The injected dopamine and octopamine solutions were prepared at a concentration of 10 μM, which is higher than the typical biological concentration of a few hundred nM measured in fly experiments. This higher concentration was chosen because, during injection, the solution diffuses and the neurotransmitter is cleared by uptake; thus, the local concentration at the electrode is much lower than 10 μM. While there is some variability in the distance between the electrode and the glass capillary, the measurements of neurotransmitter concentrations provided a wide range of concentrations for the training data set. For each electrode implantation in *Drosophila*, five injections were collected at each of five different amounts injections for both dopamine and octopamine, resulting in 25 data for each neurotransmitter. Additionally, five injections were obtained for a mixture of a dopamine and octopamine. Each electrode was postcalibrated to enable the conversion of current signals into concentration values.

### Fast-Scan Cyclic Voltammetry and Data Processing

Experimental data were acquired using the Wave-Neuro 4 potentiostat system (Pine Research, Durham, NC) and HDCV Data Acquisition Software (University of North Carolina, Chapel Hill, NC). A standard dopamine FSCV waveform (−0.4 to 1.3 V at 400 V/s, 10 Hz) was used to measure both dopamine (DA) and octopamine (OA). A two-electrode system was employed, consisting of a carbon fiber microelectrode (CFME) as the working electrode and an Ag/AgCl reference electrode. The CFME was fabricated by inserting a 7 μm carbon fiber into a glass capillary, which was pulled using a vertical electrode puller. The exposed tip was trimmed to a length of approximately 50 μm. The reference electrode was prepared by chloridizing a silver wire. A separate glass capillary was fabricated using the same pulling method as the CFME, but without inserting a carbon fiber, to deliver the neurotransmitter with the Picospritzer. The glass tip was then trimmed to a diameter of approximately 10 μm to enable localized injection near the recording site.^7^ Raw voltammogram data (time–voltage–current matrices), including DA, OA, and blank signals, were exported from HDCV in text format and imported into MATLAB for processing. Blank data were background noise recordings without neurotransmitter signals and were included during training to prevent false-positive predictions on noise-only input. Background subtraction was applied to voltammograms to remove capacitive charging currents, allowing isolation of faradaic currents associated with neurotransmitter oxidation. Data processing was performed on a workstation equipped with an AMD Ryzen 7 5700G CPU, 128 GB RAM, and an NVIDIA GeForce RTX 3050 GPU. For each data set, the standard deviation was calculated over a 3 s time window to assess signal variability. Samples with excessive noise (OA > 0.2, DA > 0.1 nA) were excluded. A sixth-order Butterworth low-pass filter (cutoff: 2000 Hz, sampling rate: 10^5^ Hz) was applied using zero-phase digital filtering to preserve signal integrity while attenuating high-frequency noise. Additionally, a 2D median filter with a 3 × 3 window size was used to remove sudden noise spikes.

### Deep Learning-Based Signal Separation

A deep learning regression model was developed in MATLAB to separate overlapping dopamine and octopamine signals. Filtered octopamine voltammograms and blank noise data were used to generate input–output pairs, with the primary oxidation peak (∼1.1 V) serving as input and the secondary peak (∼0.7 V) as output. All signals were normalized using the global mean and standard deviation, with stored parameters used for inverse normalization during inference. To improve model robustness, 5-fold time-shift augmentation was applied by shifting the signal up to 5 frames (100 ms/frame), with zero-padding to preserve alignment. Regions of interest (ROIs) were extracted from each voltammogram and resized to 128 × 128 for training. Three deep learning architectures were evaluated for this task: *U*-net, ResNet18, and LSTM. The rationale, implementation details, and performance comparison of these models are described in detail in the Results and Discussion section.

## Results and Discussion

### Signal Separation and Experimental Design

We performed in vivo neurotransmitter injection experiments in the mushroom bodies of the *D. melanogaster* brain and used the resulting data to train the machine learning network. Using the dopamine waveform for FSCV (400 V/s, from −0.4 to 1.3 V) to measure dopamine and octopamine, dopamine exhibits an oxidation peak at approximately 0.7 V and a corresponding reduction peak at around −0.2 V (Figure 1A,C). There is one oxidation and one reduction peak, and dopamine exhibits a characteristic pattern where the signal rapidly reaches a transient highest peak followed by a gradual decay (Figure 1B).^11,13^ In contrast, octopamine shows a primary oxidation peak at about 1.1 V (Figure 1D,F), followed by a relatively broad secondary oxidation peak near 0.7 V, with a reduction peak occurring at approximately −0.2 V, like that of dopamine. The first peak of OA disappears rapidly after a transient response, whereas the second peak, which is caused by a byproduct that adheres to the electrode, decays slowly (Figure 1E).^15^ The overlap of electrochemical signals at 0.7 V poses a major challenge for signal separation, especially when both neurotransmitters are detected at the same time. While principal components regression is commonly used to separate compounds with FSCV, it is only a 2D technique that assumes the CV does not change over time.^21,33,34^ Thus, this method cannot be used for separating dopamine and octopamine because the secondary oxidation peak of octopamine varies over time.

**Figure 1:**
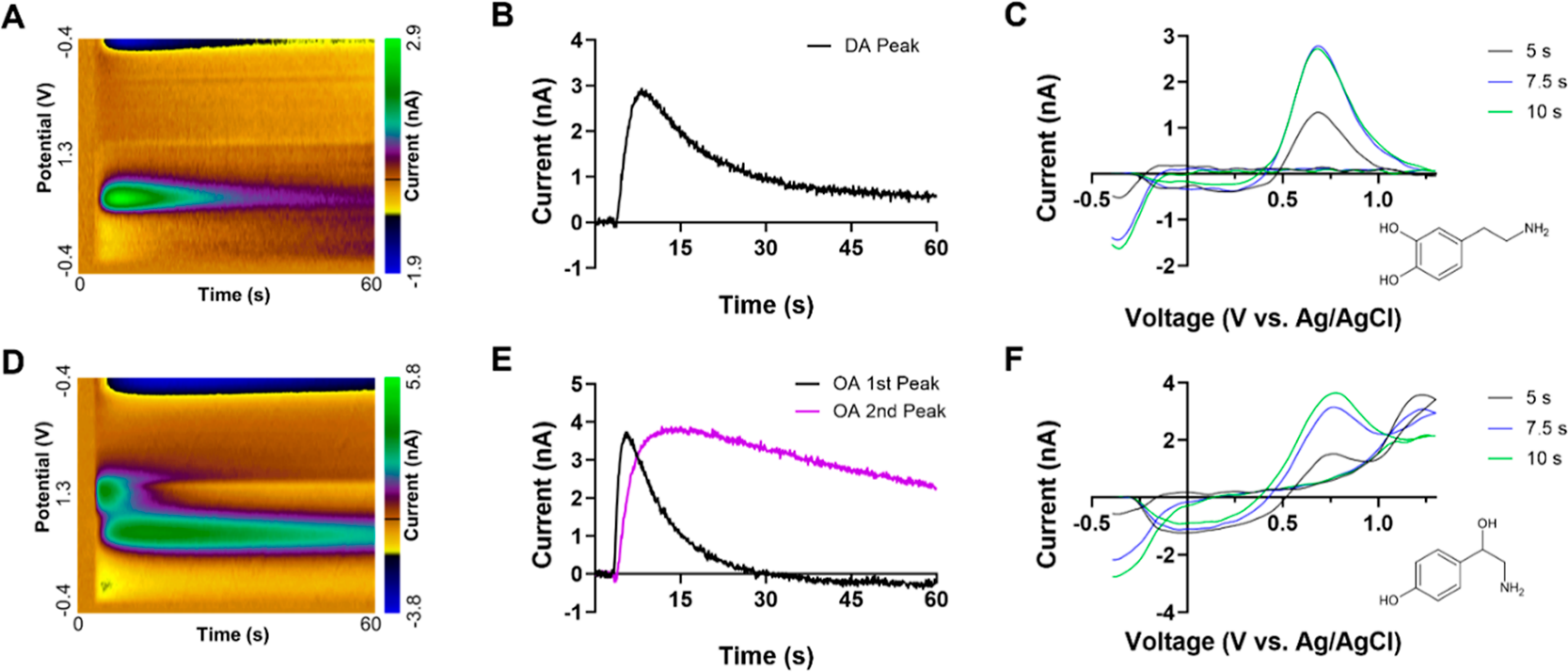
Electrochemical signals of dopamine (DA, top) and octopamine (OA, bottom), measured after injection of the analytes in the mushroom bodies of the *Drosophila melanogaster* brain. Top row: dopamine detection. (A) Color plots show current changes over time during DA, which has one oxidation and one reduction peak. In these plots, the *x*-axis represents time (s), the *y*-axis represents voltage (potential vs Ag/AgCl), and the color scale indicates current (nA), with oxidation and reduction processes appearing as distinct color bands. (B) Current vs time curves for DA. Results are background subtracted to start at 0. (C) Background-subtracted voltammograms extracted at 5, 7.5, and 10 s for DA. The inset is a molecular structure of DA. Bottom row: octopamine. (D) Color plot shows the two oxidation peaks, with the long-lasting secondary peak. (E) Current vs time traces for the primary and secondary peaks of OA. (F) Voltammograms at different time points, showing characteristic oxidation and reduction peaks. Molecular structures of OA is also shown.

Dopamine can be measured in the *Drosophila* brain and looks similar to the signal obtained in vitro in calibration experiments using flow-injection analysis. Figure 2A shows dopamine from flow-injection analysis, which has an oxidation and reduction peak for dopamine that do not change during the flow injection. Figure 2B shows a natural stimulation of dopamine in the brain, where acetylcholine is injected to cause dopamine release. The color plot shows more peak tailing and some injection errors when then acetylcholine is puffed on. This color plot looks very similar to that of dopamine when it is injected into the brain (Figure 2C).

**Figure 2:**
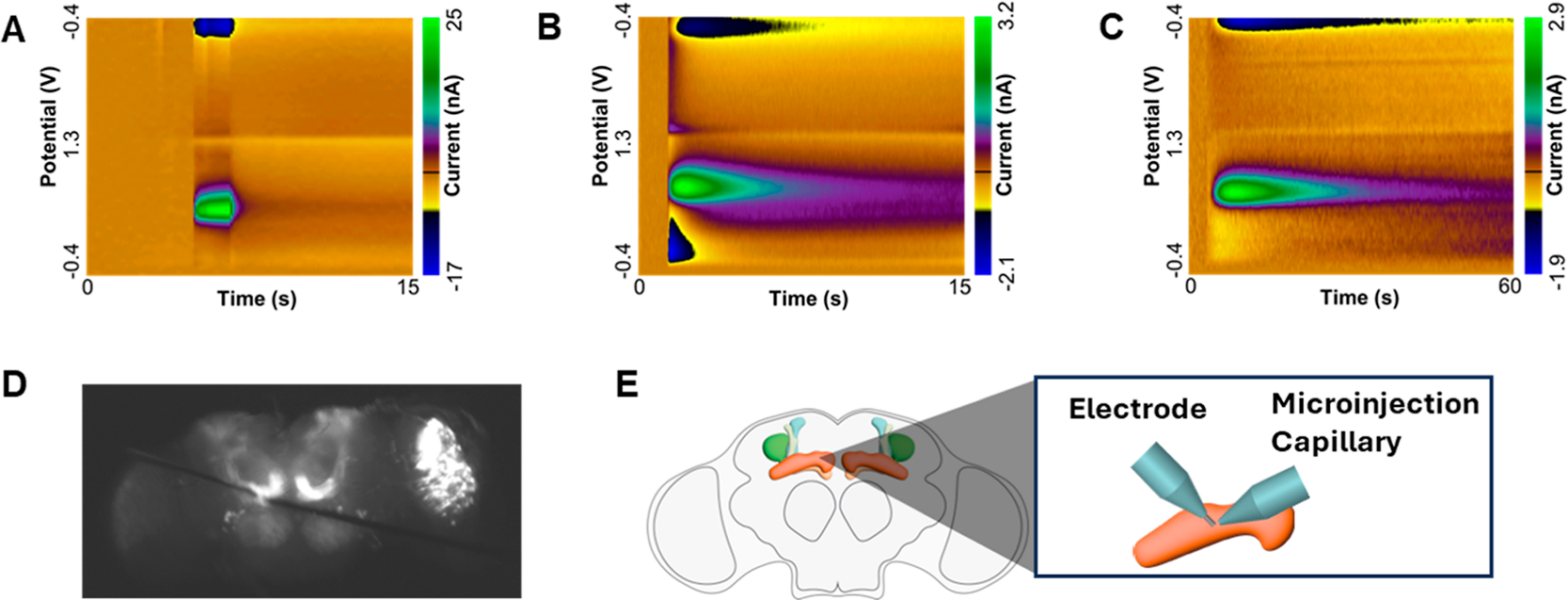
Example dopamine in *Drosophila*. (A) Dopamine measurement using a flow-injection system. (B) Acetylcholine-evoked dopamine release in the mushroom bodies of the *Drosophila* brain. (C) Dopamine response following direct dopamine injection into the brain. (D) Fluorescence image showing GFP-labeled mushroom bodies targeted by a carbon–fiber microelectrode and microinjection capillary. (E) Schematic illustration of the setup shown in (D).

The first consideration in this study was the selection of an appropriate training set. While using flow injection data from in vitro calibrations would be easiest, the color plots do not look as similar to data collected in tissue. Flow injection has unrestricted diffusion, no protein or tissue that sticks to the electrode, and faster kinetics of washout, rather than reuptake.^7,13^ As a result, the three-dimensional color plots of time, voltage, and current generated from flow injection data differ in shape from those obtained from evoked dopamine release measurements. We chose to use data obtained from experiments in which neurotransmitters were measured after injection into the brains of fruit flies. In this experiment, a carbon–fiber microelectrode (CFME) and a sharpened glass capillary filled with a neurotransmitter solution are positioned in the medial lobe of mushroom bodies (Figure 2D,E). The neurotransmitter solution is pressure-injected, and its concentration in the extracellular space is monitored using fast-scan cyclic voltammetry (FSCV). This leads to color plots that more closely resemble acetylcholine-evoked dopamine release data (Figure 2B,C),^6,7^ and have the same diffusion and uptake profiles because they are in tissue. Previous studies that employed principal component regression (PCR) for FSCV signal analysis consistently used in vivo data for training to predict in vivo signals, highlighting the importance of using training data collected under the same experimental conditions, such as electrode type, buffer composition, and biological environment, as the target data.

In this study, we propose a method that defines regions of interest (ROIs) near the primary and secondary oxidation potentials (Figure 3A). In this approach, the primary oxidation peak is used as the input and the secondary peak as the output for training a deep learning network. Because the primary oxidation potential of octopamine is approximately 1.1 V and does not overlap with the oxidation or reduction peaks of dopamine, it can be assumed that, in a mixture, the signal at 1.1 V originates solely from octopamine. The secondary oxidation peak of octopamine is predicted from the primary peak, and its contribution can then be subtracted from the mixed signal. This enables the estimation of dopamine oxidation peaks in mixtures.

**Figure 3:**
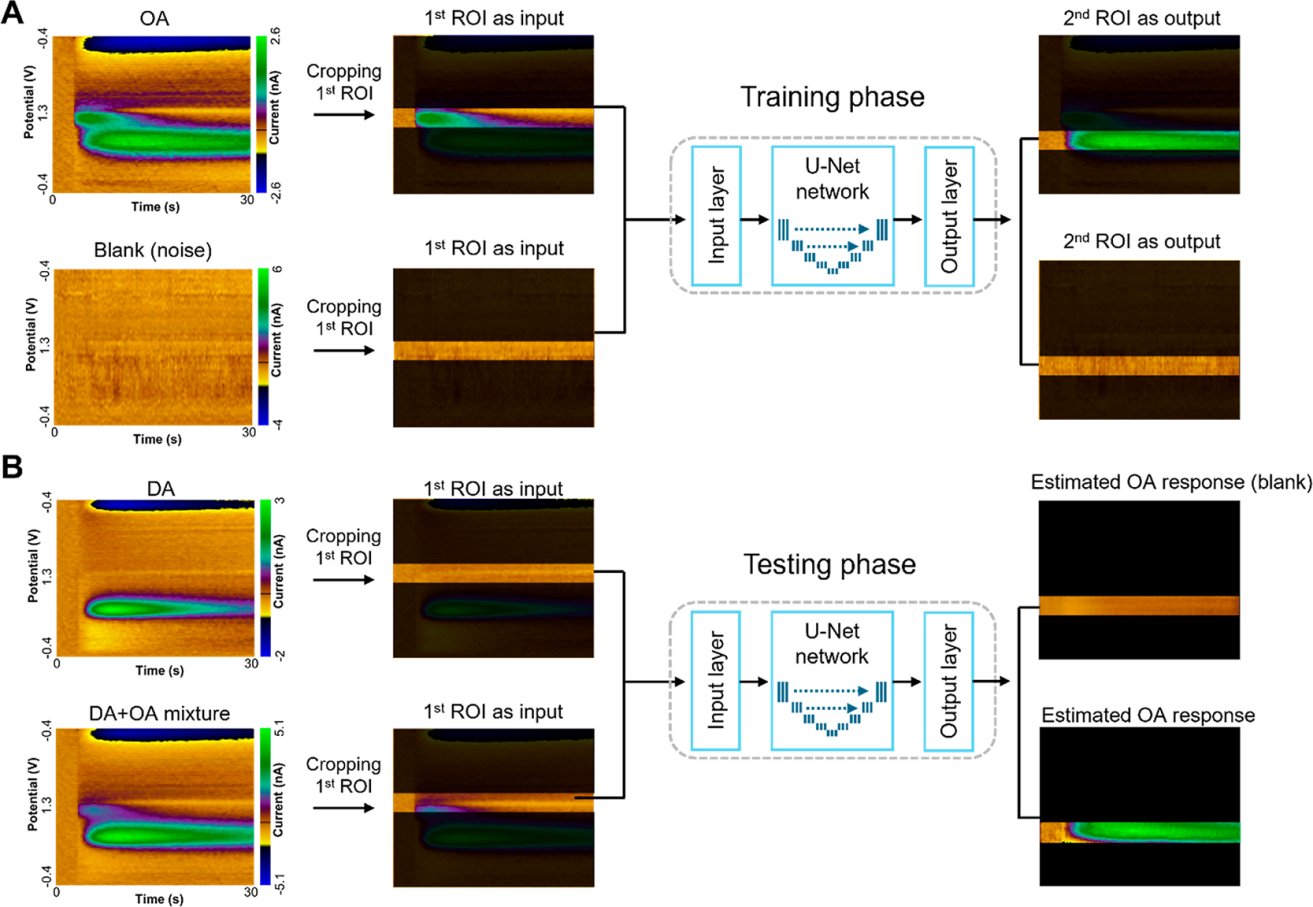
Example of constructing a regression network using a *U*-net architecture. (A) The network was trained using octopamine data and blank (no-signal) data. The first ROI was used as input and the second ROI as the output target. (B) For validation, dopamine or dopamine–octopamine mixtures were used. The first ROI was provided as input to the network, and the second ROI was predicted. Primary-to-secondary localized regression.

A key consideration in this process is the nature of carbon–fiber microelectrodes, which are handcrafted and therefore exhibit variability in impedance. This variability can lead to slight shifts in the measured oxidation potentials, making it essential to precisely define the ROIs corresponding to the primary and secondary peaks of octopamine for each electrode. To address this, we adopted an in situ prediction method based on the background capacitive current, as proposed by the Sombers group.^35^ Specifically, the position of the quinone peak, which results from the oxidation of quinone-like groups on the CFME surface, was identified and used to predict the location of octopamine’s oxidation peaks via linear regression. The predicted values were then used to define the center of each ROI window, with ±50 data points selected in the voltage dimension to yield a 100-point window (Figure S1). Each FSCV scan generates a voltammogram consisting of 850 data points (100 kHz sampling rate, 8.5 ms scanning time). Considering the broadness of octopamine oxidation peaks, the ROI was set to 100 data points in the voltage axis. Although the full data set spans 600 time points (10 Hz scanning for 60 s), the time window was limited to 300 time points to capture the main decay pattern of dopamine and octopamine responses. Consequently, each ROI was defined as a 300 × 100 matrix (300 time points × 100 voltage–current points). The ROI around the primary oxidation peak was designated as the first ROI (input), and the ROI around the secondary peak as the second ROI (output).

For data acquisition, a carbon–fiber microelectrode and a neurotransmitter-injecting glass capillary were positioned at either the heel or medial tip of the mushroom body (MB) to simulate in vivo neurotransmitter release. A total of 226 color plots (206 octopamine injections and 20 blanks), which passed the noise exclusion criteria, were collected from 11 electrodes. These data were used to train the deep learning network, with the region corresponding to the first ROI serving as the input and that of the second ROI as the output (Figure 3A).

After training, the network was tested using data from either DA alone or DA–OA mixtures. As in the training phase, the first ROI of dopamine or the DA–OA mixture was provided as input to the network to predict the OA second oxidation peak response from the input (Figure 3B). In the case of dopamine alone, there was no significant signal in the first ROI for octopamine, and accordingly, the network did not produce any meaningful output. In contrast, for the mixture data, to recover the original dopamine signal, the predicted OA second oxidation response was subtracted from the second ROI of the mixture data, allowing estimation of the dopamine component.

### Deep Learning Model Architecture and Training

We evaluated three deep learning architectures: *U*-net, ResNet18, and LSTM to resolve and quantify dopamine and octopamine signals from the time–voltage–current data set (Supporting Information Figures S2 to S4). Given the modest data set size, we selected architectures known to perform well in small-data settings, such as LSTM, as well as ResNet and *U*-net, which have been successfully adapted to such conditions and can capture both spatial and temporal patterns.^36–38^ Each model was trained using hyperparameters individually optimized for its architecture, including the number of training epochs, batch sizes, and other settings to ensure optimal performance. To evaluate generalization performance, we computed the normalized root-mean-square error (NRMSE) on a held-out test set comprising unknown mixture data not used during model optimization. The NRMSE was calculated as the difference between the estimated dopamine signal from the mixture input and the ground truth dopamine component. Among the tested architectures, *U*-net achieved the lowest NRMSE and produced results that were visually the most consistent with the target outputs, making it the most suitable model for this application (Supporting Information Figures S2–S4).

The *U*-net was configured with an input size of 128 × 128 × 1 and an encoder depth of 4. Although the original input and output data were shaped as 300 × 100, both were resized to 128 × 128 to match the network configuration. After inference, the predicted output was rescaled back to 300 × 100 to match the original resolution. Although *U*-net was initially designed for segmentation tasks, it was modified for regression in this study. Specifically, the final convolutional layer was adjusted to produce a single output channel, and the segmentation layer was replaced with a regression layer. The softmax layer was removed, and the final convolutional layer was directly connected to the regression output. The model was trained for up to 40 epochs with a mini-batch size of 16. A piecewise constant learning rate schedule was employed, reducing the learning rate by half every 10 epochs to ensure stable convergence.

Brief descriptions of the LSTM, ResNet18, and *U*-net models are provided in Supporting Information Figures S2–S4, along with representative results from 16 sample cases for each model to illustrate the visual differences in their outputs. These examples highlight performance characteristics not fully captured by quantitative metrics, such as artifact suppression, spatial consistency, and peak shape accuracy. While the normalized root-mean-square error (NRMSE) values were similar across models, qualitative differences were evident. The LSTM model occasionally exhibited instability in spatial consistency due to its sequence-based architecture. The ResNet18 model, although more spatially stable, produced low-resolution outputs with staircase-like artifacts and unintended signals in noise-only regions. In contrast, the *U*-net model preserved spatial detail and temporal coherence more effectively, without introducing such artifacts. These advantages likely stem from the skip-connection structure of *U*-net, which allows fine-grained information from earlier layers to be retained during reconstruction. Supporting Information Figures S2–S4 thus serve as important qualitative validation, complementing the quantitative evaluation. They provide visual evidence of artifact patterns, inconsistencies, and reconstruction quality that may not be fully reflected in RMSE alone. Based on these observations, *U*-net was selected as the final model in this study.

### Network Validation and Mixture Analysis

Given the relatively small data set size (1130 samples after 5-fold augmentation, including 1030 neurotransmitter samples and 100 blank samples), there was a risk of overfitting, which could compromise generalization to unseen data. To mitigate this, we employed 5-fold cross-validation to rigorously evaluate the model’s generalization performance. The data set was randomly partitioned into five equal subsets, with four subsets used for training and one for validation in each iteration, ensuring that every sample served as validation data exactly once. For each fold, the normalized root-mean-square error (NRMSE) was calculated for both the training and validation sets based on the z-score normalized data. On average, the training NRMSE was 0.097 ± 0.10 and the validation NRMSE was 0.082 ± 0.07 across the five folds, indicating consistent model performance without signs of overfitting. This trend is further supported by Figure S6, which shows that excluding the test electrode during training increased average NRMSE from 0.078 to 0.120 for dopamine and from 0.092 to 0.140 for octopamine (*n* = 8, paired *t*-test, *p* < 0.05), reflecting moderate but acceptable degradation in performance under more stringent testing conditions. These findings support the robustness of the model and its ability to generalize across different electrodes.

To further validate the model’s generalization beyond cross-validation, we evaluated its performance on data distinct from the training distribution. First, we tested computational mixtures (Figure 4A), was generated in silico by mathematically combining independent DA and OA recordings. To further validate the model’s generalization beyond cross-validation, we tested the network using two types of dopamine–octopamine mixtures: computational mixtures, generated in silico by combining individual DA and OA recordings, and experimentally prepared mixtures, created by physically coinjecting DA and OA solutions into the brain (Figure 4A). A key advantage of using computational mixtures is the availability of ground truth DA and OA components, which enables quantitative evaluation of signal separation. The algorithm was designed to predict the OA component within the second ROI of a mixture, using the first ROI as input. By subtracting the predicted OA signal from the original mixture, the DA component was also recovered (Figure 4B,D). Visual inspection of the color plots and time courses of peak currents (Figure 4C,E) further supported the predictive capability of the network. To quantitatively evaluate the separation performance, the original OA and DA signals used to generate the mixtures were treated as ground truth references, allowing for the calculation of NRMSE for each component. The proposed algorithm achieved an NRMSE of 0.06 for dopamine and 0.08 for octopamine, indicating high separation accuracy with errors well below 10%. These results demonstrate the effectiveness and reliability of the proposed approach in decomposing overlapping neurochemical signals.

**Figure 4:**
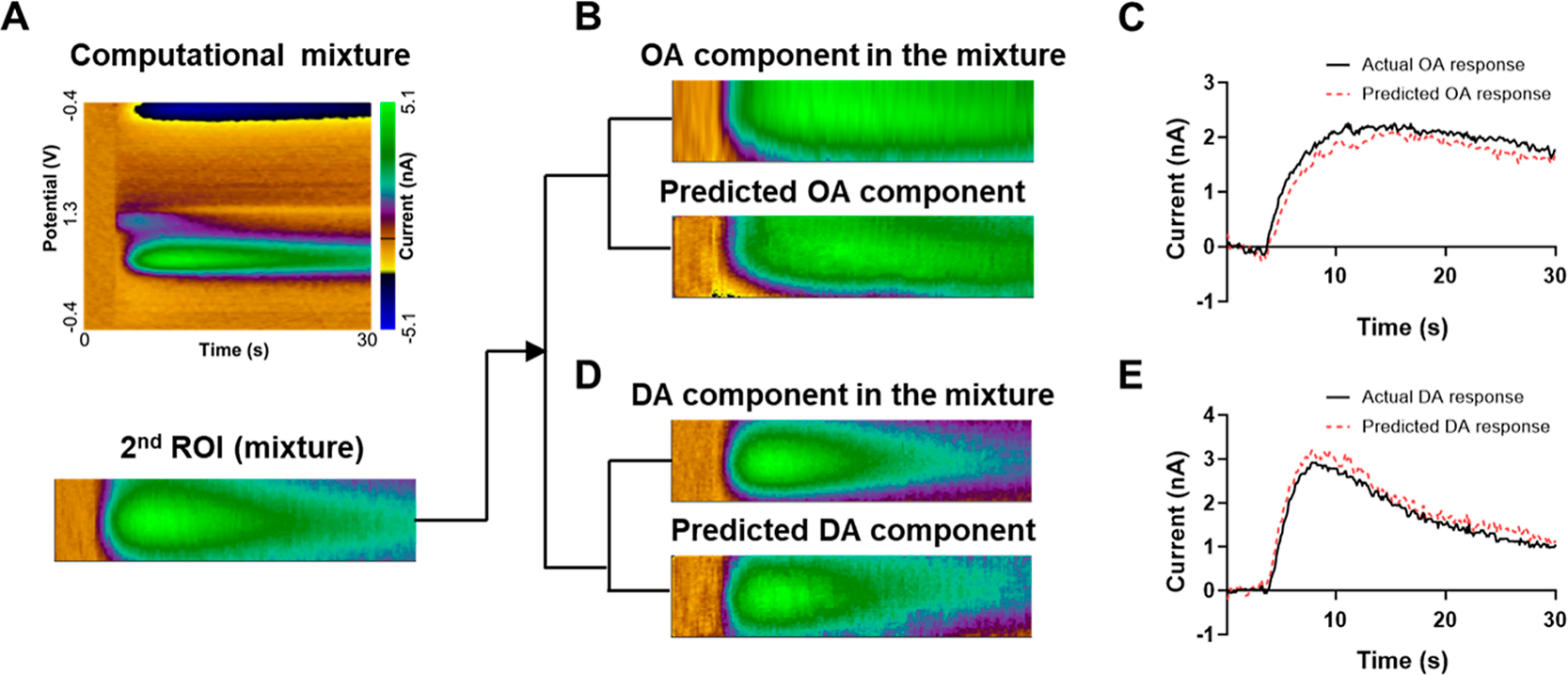
Decomposition of a computational mixture using the *U*-net regression network. (A) Original plot, and input color plot of the mixture. (B) (top) The OA data used to construct the mixture. (bottom) The second oxidation response of octopamine (OA) was predicted from the first ROI using the trained network. (C) Time course of the predicted OA peak current (red dashed line is predicted response, black line is actual DA response). (D) (top) Dopamine component in the mixture. (bottom) Dopamine (DA) signal was obtained by subtracting the predicted OA response (B) from the second ROI of the mixture. (E) Time course of the DA peak current.

After validating the model on computational mixtures, additional testing was conducted using experimentally measured mixtures composed of dopamine and octopamine at a 1:1 concentration ratio (Figure 5). Unlike the computational mixtures, the experimental mixtures did not provide access to ground truth signals, making it infeasible to perform numerical comparisons with the true dopamine or octopamine components. Therefore, the quality of decomposition was evaluated by comparing the predicted signals to the reference recordings using both visual assessment and quantitative similarity metrics. Structural similarity index (SSIM) values were calculated between the predicted and reference color plots, yielding 0.77 ± 0.06 for dopamine and 0.75 ± 0.10 for octopamine, indicating high structural agreement despite biological variability (Figure S5A,B). When injecting a mixture into the brain, it was necessary to replace the injecting capillary with one filled with the mixture solution. As a result, slight variations in the measured concentration and local environment were inevitable due to differences in the distance between the electrode and the newly positioned injecting capillary, so there is no ground truth for comparison. To quantify the predicted concentrations of dopamine and octopamine from the mixtures, postcalibration data obtained from pure analytes was applied to the separated signals. Because each mixture was prepared with a 1:1 concentration ratio of dopamine and octopamine, the predicted values were expected to lie along the identity line (*x* = *y*). Accordingly, a scatter plot was used to assess the agreement between the predicted peak currents of each component, revealing a strong correlation (Pearson’s *r* = 0.929, concordance correlation coefficient (CCC) = 0.925) across 25 mixture samples obtained from four electrodes (Figure 5E). To further evaluate agreement beyond correlation alone, a Bland–Altman analysis was performed. This method accounts for potential systematic biases even when correlation is high and showed that most data points fell within the 95% limits of agreement (±1.96 SD), supporting consistent performance of the model across samples (Figure 5F). These results demonstrate that the proposed network successfully separated dopamine and octopamine components in both computational and experimentally measured mixtures, with prediction errors remaining within an acceptable noise range.

**Figure 5:**
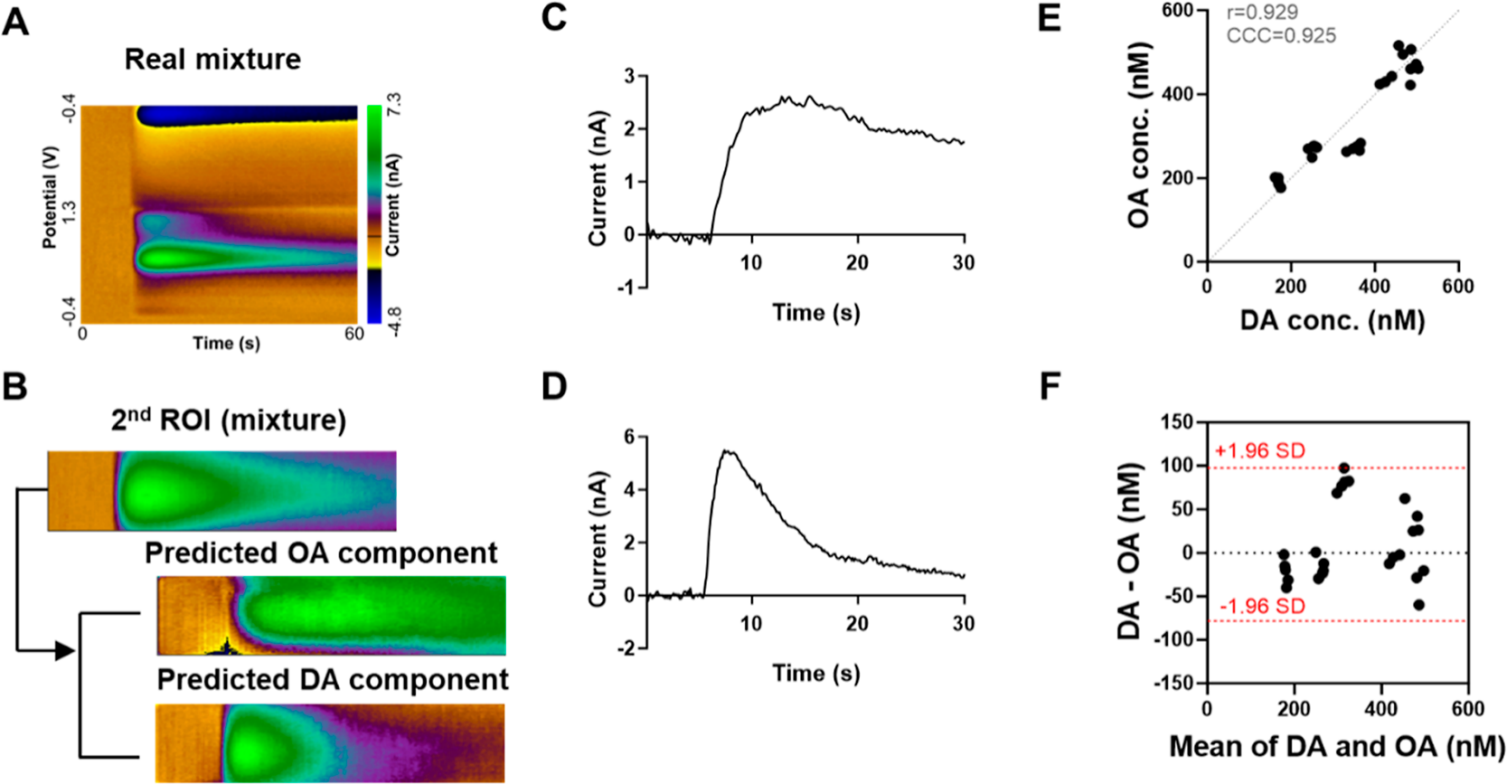
Decomposition of an experimentally measured mixture using the *U*-net regression network. (A) Original color plot of the DA–OA mixture. (B) Second ROI of the mixture, along with predicted octopamine (OA) and dopamine (DA) components. (C) Time course of the predicted OA second oxidation peak. (D) Time course of the predicted DA oxidation peak. (E) Scatter plot comparing predicted OA and DA concentrations from 25 mixture samples (from 4 electrodes). Each mixture had a 1:1 concentration ratio between DA and OA. (F) Bland–Altman plot showing the agreement between predicted DA and OA concentrations, with most data points falling within the 95% limits of agreement (±1.96 SD).

### Toward Application to Natural Signals

This work demonstrates that the model successfully separates dopamine and octopamine components in real, experimentally measured mixtures collected from the *Drosophila* brain. These results demonstrate the feasibility of applying deep learning–based voltammetric analysis to biologically relevant data. The network accurately decomposed overlapping signals even in complex tissue environments, enabling reliable quantification of both neurochemicals. Although variability between electrodes is a known limitation in FSCV, the model maintained reasonable accuracy when evaluated on unseen electrodes, as detailed in Supporting Information Figure S6. Moving forward, this approach lays the groundwork for application to natural signals, such as behaviorally evoked dopamine and octopamine release during learning. By enabling simultaneous, artifact-resistant detection of multiple neuromodulators, this method provides a powerful tool for probing the neural mechanisms underlying reinforcement and memory in *Drosophila*.

## Conclusions

We developed a modified *U*-net–based regression model to separate overlapping dopamine (DA) and octopamine (OA) signals in fast-scan cyclic voltammetry (FSCV). Trained on in vivo injection data, the model accurately predicted the secondary OA peak from its primary oxidation signal and subtracted it from the mixture to isolate the DA component. Validation with computationally synthesized mixtures yielded NRMSE values below 10%, demonstrating high signal separation accuracy. When applied to experimentally measured DA–OA mixtures, the model achieved strong agreement between predicted DA and OA concentrations (*r* = 0.93, CCC = 0.93) and high structural similarity (SSIM ≈ 0.76), despite biological variability. The model also maintained acceptable performance on data from electrodes excluded during training, with prediction errors typically under 15%. These results highlight the model’s effectiveness for resolving overlapping, time-varying voltammetric signals and suggest broader applicability to other dynamic electrochemical sensing challenges in the *D. melanogaster* brain.

## Supplementary Material



## Notes

Figure S1 correlation of quinone-like groups and octopamine oxidation peaks. Comparison of quinone-like groups oxidation peaks for predicting octopamine oxidation peaks. Figure S2 LSTM-based regression network. Performance evaluation and identification of visual artifacts and noise in the LSTM model. Figure S3 ResNet 18-based regression network. Performance analysis and observation of upsampling artifacts and spurious early signals. Figure S4 *U*-net-based regression network. Final model selection based on NRMSE performance, visual consistency, and mitigation of low-resolution artifacts. Figure S5 predicted color plots. Structural similarity analysis between real mixture separation and pure analyte samples. Figure S6 leave-one-electrode-out validation. Assessment of NRMSE changes and electrode-specific feature reliance upon electrode exclusion (PDF)
