# Supplementary material for: Machine Learning for Separating Dopamine and Octopamine Electrochemical Signals in Drosophila
Source: Anal Chem. 2026 Jan 2;98(1):249–57. doi: 10.1021/acs.analchem.5c04155 (PMC12809646; doi:10.1021/acs.analchem.5c04155)
Supplement: Supplementary file 1 [file ac5c04155_si_001.pdf]

Supporting Information

Machine Learning For Separating Dopamine and Octopamine Electrochemical signals in Drosophila

Cheonho Park, B. Jill Venton*

Department of Chemistry, University of Virginia, PO Box 400319, Charlottesville, Virginia 22904, United States

Figure S-1. Correlation of Quinone-like Groups and Octopamine Oxidation Peaks............S-3

Figure S-2. LSTM-Based Regression Network..S-4

Figure S-3. ResNet18-based Regression Network...S-6

Figure S-4. U-Net-based Regression Network...S-8

Figure S-5. Predicted color plots..S-10

Figure S-6. Leave-One-Electrode-Out validation..S-11

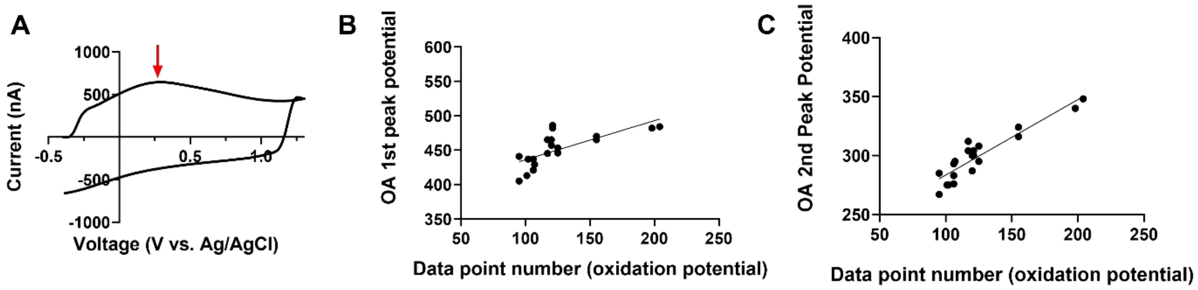

Figure S-1. (A) Background current measured by FSCV showing the oxidation peak of quinone-like groups, analyzed in relation to octopamine oxidation potentials. (B) Correlation between quinone-like oxidation potential (point number in voltammogram) and octopamine's first oxidation peak (point number in voltammogram). (C) Correlation between quinone-like oxidation potential (point number in CV) and octopamine's second oxidation peak (point number in CV). For clarity, the x- and y-axes in (B) and (C) are shown in data point index rather than voltage, as cyclic voltammetry produces overlapping potential values during the forward and backward scans, making it difficult to uniquely identify peak positions using voltage alone.

S-1. Correlation of Quinone-like Groups and Octopamine Oxidation Peaks

In a study by the Sombers group[1], it was demonstrated that the shape of the capacitive current, particularly the peak region, recorded during FSCV (Fast-Scan Cyclic Voltammetry) with carbon fiber microelectrodes (CFMEs) can be used to estimate the oxidation peak potential of dopamine. Due to the hand-crafted nature of CFMEs, dopamine peak potentials vary between electrodes. To account for this variability, the study proposed an algorithm to estimate peak shifts using the oxidation peak of quinone-like group. In the present work, we reproduced this approach and examined the relationship between the capacitive current peak potential and the primary and secondary oxidation peaks of octopamine. Linear regression yielded R^2 values of 0.4955 for the primary peak and 0.8453 for the secondary peak. The estimates obtained through linear regression were directly used to define the first and second ROIs corresponding to the primary and secondary oxidation peaks, as described in the main manuscript. While more sophisticated peak-finding algorithms have been developed and could have been applied to further refine the estimates, such methods generally assume the presence of distinct peak features and were considered unnecessarily complex for this purpose. Instead, the predicted ROIs were visually examined and found to reliably contain the relevant peaks. Each ROI covered a sufficiently wide range (approximately 100 out of 850 whole potential data points), and no cases were observed where dopamine or octopamine peaks fell outside of these regions.

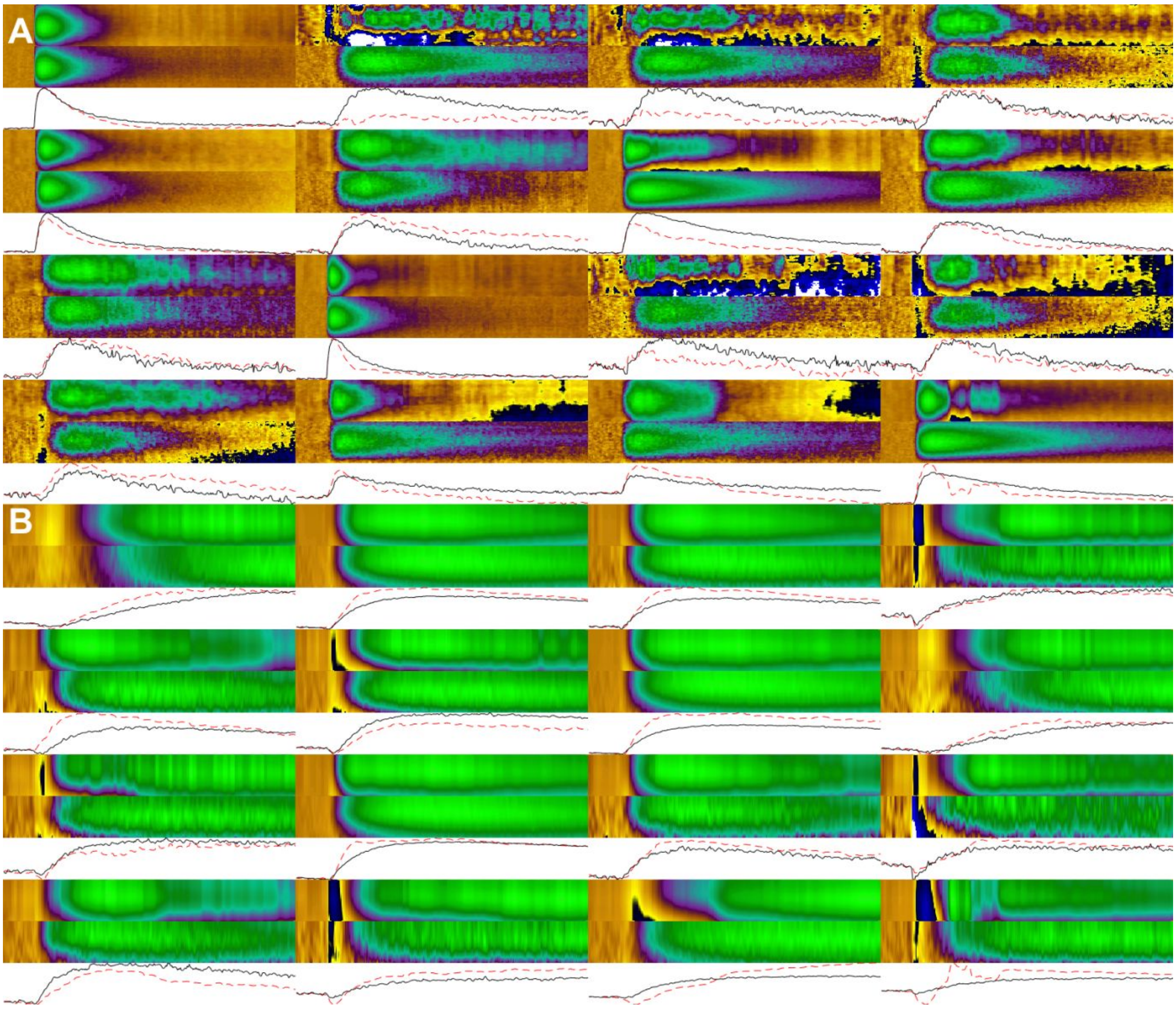

Figure S-2. Visualization of dopamine and octopamine prediction results using an LSTM-based model for computationally synthesized mixtures of dopamine and octopamine. The figure includes two sets of 16 example color plots: (A) dopamine predictions and (B) octopamine predictions. For each grid: the top color plot is the (1) the predicted dopamine (set A) or octopamine (set B) component extracted from the mixture by the model, and the bottom color plot is (2) the ground truth dopamine data (set A) or the ground truth octopamine data (set B) used for training. Under each pair of color plots are the representative current–time (i vs. t) curve at the dopamine oxidation peak potential. The red dotted line is the model prediction, while the black line shows the ground truth. The synthetic mixtures were created by linearly combining dopamine and octopamine voltammograms.

S-2. LSTM-Based Regression Network

An LSTM-based regression model was implemented to predict the second oxidation voltammogram from the first. The network comprised a sequence input layer, an LSTM layer with 50 hidden units, a dropout layer (rate = 0.5), a fully connected layer, and a regression layer. Input and target signals were normalized using global statistics, and noise-augmented samples were included to improve generalization. The model was trained for 200 epochs using the Adam optimizer (learning rate = 1×10^{-3}) with early stopping based on validation performance. Training completed in 403 seconds. After training, predictions on synthetic mixtures were inverse-normalized for evaluation. The model achieved normalized root mean square error (NRMSE) values of 0.0652 for dopamine and 0.0846 for octopamine. However,

visual inspection revealed artifacts and noise in some outputs (Fig. S2). Fig. S2 shows the data from computational mixtures of dopamine and octopamine, showing 16 electrodes comparing the color plots for the model output and ground truth, and then the I vs t. Visual inspection reveals some disotrtions and artifacts, despite the overall high NRMSE. These issues persisted despite hyperparameter tuning, indicating limitations of the current architecture. An alternative model was therefore explored to improve robustness.

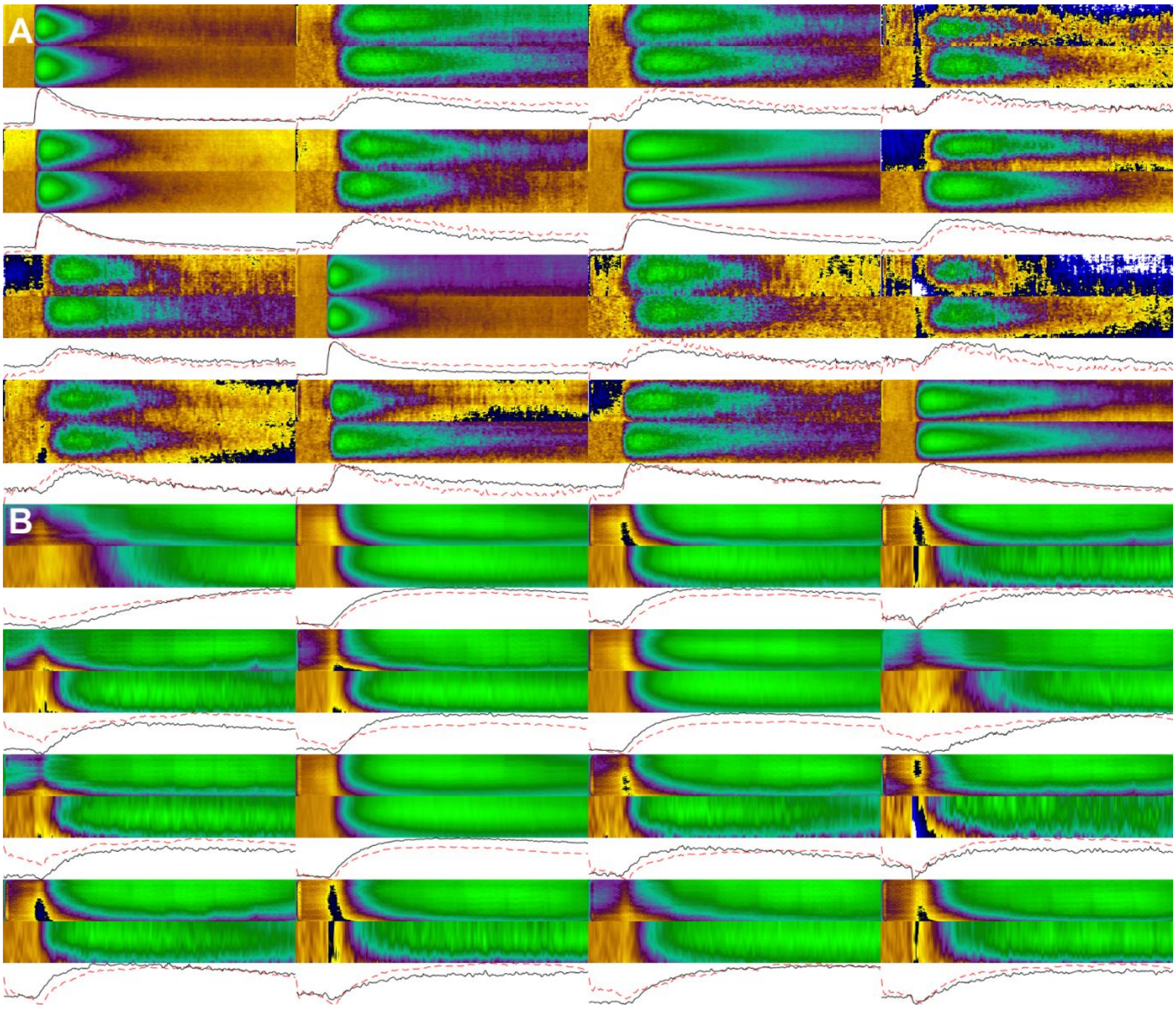

Figure S-3. Visualization of dopamine and octopamine prediction results using an ResNet-based model for computationally synthesized mixtures of dopamine and octopamine. The figure includes two sets of 16 example color plots: (A) dopamine predictions and (B) octopamine predictions. For each grid: the top color plot is the (1) the predicted dopamine (set A) or octopamine (set B) component extracted from the mixture by the model, and the bottom color plot is (2) the ground truth dopamine data (set A) or the ground truth octopamine data (set B) used for training. Under each pair of color plots are the representative current–time (i vs. t) curve at the dopamine oxidation peak potential. The red dotted line is the model prediction, while the black line shows the ground truth. The synthetic mixtures were created by linearly combining dopamine and octopamine voltammograms.

S-3. ResNet18-based Regression Network

A ResNet18-based regression model was implemented to predict the second oxidation voltammogram from the first. The model was adapted from ResNet-18 by removing its final classification layers and appending a sequence of transposed convolutional layers for multi-stage upsampling, progressively restoring the spatial resolution to the original input size. A final regression layer generated the output voltammogram. Input and target data were normalized using global statistics and resized to 224×224. Grayscale inputs were converted to pseudo-RGB by replicating the single channel across three channels. The model was

trained for 40 epochs using the Adam optimizer (initial learning rate = 1×10^{-4}), with a piecewise learning rate schedule, L2 regularization, and early stopping based on validation loss. Training was completed in 369 seconds. After training, predictions on synthetic mixtures were inverse-normalized for evaluation. The model achieved normalized root mean square error (NRMSE) values of 0.0604 for dopamine and 0.0786 for octopamine, outperforming the LSTM-based model. Fig. S3 shows the data from ResNet for computational mixtures of dopamine and octopamine, showing 16 electrodes comparing the color plots for the model output and ground truth, and then the I vs t. Visual inspection confirmed reduced artifacts and improved spatial coherence, suggesting that the ResNet-based architecture with multi-stage upsampling provided a more robust solution. However, since ResNet was originally designed for classification, the network inherently compresses spatial resolution in its intermediate layers. As a result, despite the added upsampling stages, the predicted octopamine voltammograms exhibited a visibly upsampled appearance with staircase-like artifacts in the predicted octopamine color plots. In addition, unintended signals occasionally appeared in early blank regions (e.g., the first 5 seconds), which should contain only noise due to the background subtraction process. This may be attributed to the loss of spatial precision caused by low-resolution feature representations, which can hinder the model's ability to distinguish between signal and background in temporally sparse regions.

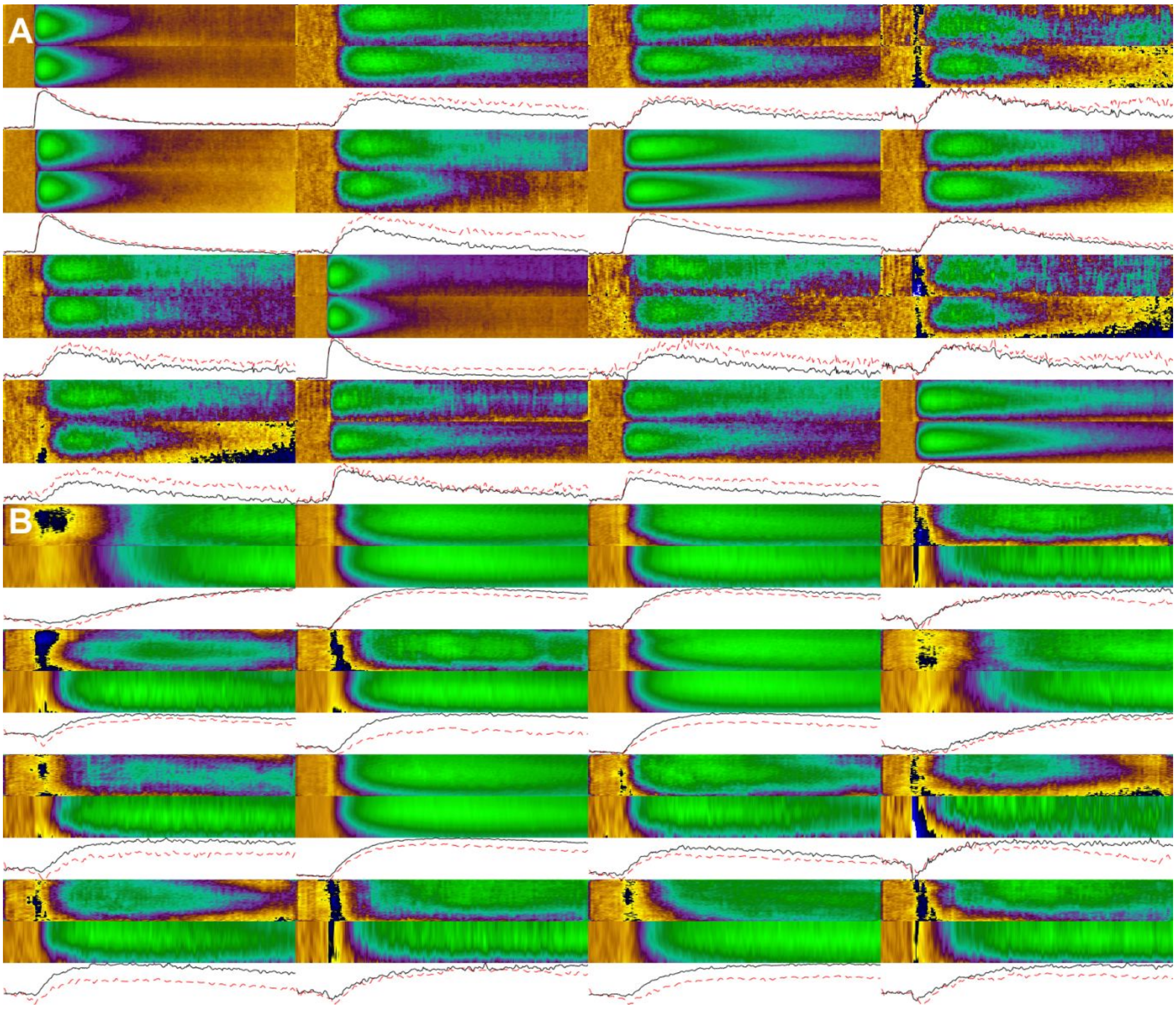

Figure S-4. Visualization of dopamine and octopamine prediction results using an U-Net-based model for computationally synthesized mixtures of dopamine and octopamine. The figure includes two sets of 16 example color plots: (A) dopamine predictions and (B) octopamine predictions. For each grid: the top color plot is the (1) the predicted dopamine (set A) or octopamine (set B) component extracted from the mixture by the model, and the bottom color plot is (2) the ground truth dopamine data (set A) or the ground truth octopamine data (set B) used for training. Under each pair of color plots are the representative current–time (i vs. t) curve at the dopamine oxidation peak potential. The red dotted line is the model prediction, while the black line shows the ground truth. The synthetic mixtures were created by linearly combining dopamine and octopamine voltammograms.

S-4. U-Net-based Regression Network

A U-Net-based regression model was implemented to predict the second oxidation voltammogram from the first. The architecture was adapted from the standard U-Net with an encoder depth of five and an increased number of initial filters (64). The final segmentation layers were replaced with a single-channel convolutional layer and a regression layer to support continuous output. The softmax layer was removed. Input and target voltammograms were normalized using global statistics and resized to 128×128. Temporal shift-based data augmentation was applied to improve robustness and generalization. The model was trained

for 100 epochs using the Adam optimizer (initial learning rate = 1×10^{-4}), with a piecewise learning rate schedule, L2 regularization, and early stopping based on validation performance. Training was completed in 1596 seconds. After training, predictions on synthetic mixtures were inverse-normalized for evaluation. The model achieved normalized root mean square error (NRMSE) values of 0.0610 for dopamine and 0.0795 for octopamine. Compared to the LSTM- and ResNet-based models, the U-Net architecture demonstrated improved visual consistency and accuracy, suggesting better spatial learning and generalization to unseen mixture patterns. Fig. S4 shows the data from computational mixtures of dopamine and octopamine, showing 16 electrodes comparing the color plots for the model output and ground truth, and then the I vs t traces. Although the NRMSE values of the ResNet- and U-Net-based models were comparable, a qualitative comparison revealed notable differences. The U-Net model, which incorporates skip connections between encoder and decoder paths, effectively preserved spatial detail and mitigated the low-resolution artifacts observed in the ResNet outputs. In particular, the U-Net predictions did not exhibit the staircase-like patterns or spurious early signals occasionally seen in the ResNet results. In addition to its structural advantage, U-Net is well-suited for data with continuous spatial or temporal features, such as FSCV voltammograms, where preserving temporal consistency is critical. Its ability to retain fine-grained information and maintain continuity across frames contributed to more stable and realistic outputs. Accordingly, the U-Net architecture was selected as the final model in this study.

A

B

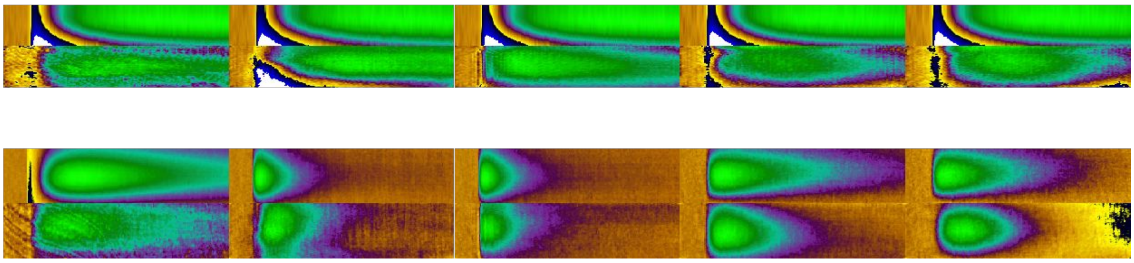

Figure S-5. Predicted 2nd oxidation color plots of (A) octopamine and (B) dopamine. In each panel, the top row shows real octopamine/dopamine samples with similar patterns, while the bottom row shows the predicted octopamine/dopamine. The structural similarity index (SSIM) obtained from a total of 25 mixture samples (acquired from 4 different electrodes) was 0.77 ± 0.06 for dopamine and 0.75 ± 0.10 for octopamine.

S-5. Predicted color plots.

Since ground truth is not available for real mixture data, this study employed alternative validation methods. One such method was the use of the structural similarity index (SSIM). SSIM yields a value close to 1 when two images are highly similar and approaches 0 when they are dissimilar. The predicted dopamine and octopamine samples were compared with real pure dopamine/octopamine samples in the same Drosophila nerve cord, and the highest SSIM value among them was selected as the SSIM for each mixture (Fig. S5).

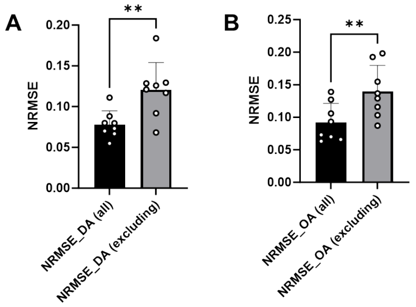

Figure S-6. Comparison of normalized root mean square error (NRMSE) when the test electrode was included (All) or excluded (Excl.) during training. (A) Dopamine: average NRMSE increased from 0.078 (All) to 0.120 (Excl.). (B) Octopamine: average NRMSE increased from 0.092 (All) to 0.140 (Excl.). Each dot represents an individual electrode, and bars indicate the group mean. Statistical significance was determined by paired t-test ($n = 8$, $p < 0.05$).

S-6. Leave-One-Electrode-Out validation.

To assess the generalization performance and robustness of the proposed U-Net-based regression network across different electrodes, we conducted a leave-one-electrode-out experiment. In this approach, data from one electrode were excluded during training and used only for testing. This procedure was repeated for all ten electrodes. For each electrode, we compared two conditions: one trained with all electrodes (including the test electrode) and another trained with all except the test electrode. In both cases, testing was performed on the excluded electrode. As shown in Figure S6, the NRMSE for both dopamine (A) and octopamine (B) increased significantly when the test electrode was excluded from training. On average, the NRMSE for dopamine increased from 0.078 to 0.120, and for octopamine from 0.092 to 0.140. A paired t-test ($n = 8$) confirmed that these differences were statistically significant ($p < 0.05$), indicating that the model relies, to some extent, on electrode-specific features. Nevertheless, the prediction errors in the exclusion condition remained mostly within 15%, suggesting that the model retains practically acceptable performance despite the absence of test electrode data. This highlights the model's partial robustness to electrode variability and suggests that generalization can be further improved through the inclusion of more diverse electrode data or domain adaptation strategies.

1. Meunier, C. J.; Roberts, J. G.; McCarty, G. S.; Sombers, L. A., Background signal as an in situ predictor of dopamine oxidation potential: improving interpretation of fast-scan cyclic voltammetry data. *ACS chemical neuroscience* **2017,** *8* (2), 411-419.
